# Exploring social inequalities in healthcare trajectories following diagnosis of diabetes: a state sequence analysis of linked survey and administrative data

**DOI:** 10.1186/s12913-021-07450-9

**Published:** 2022-01-31

**Authors:** Rachel McKay, Laurence Letarte, Alexandre Lebel, Amélie Quesnel-Vallée, on behalf of the TORSADE Cohort Working Group, Alain Vanasse, Gillian Bartlett, Lucie Blais, David Buckeridge, Manon Choinière, Catherine Hudon, Anaïs Lacasse, Benoit Lamarche, Alexandre Lebel, Amélie Quesnel-Vallée, Pasquale Roberge, Valérie Émond, Marie-Pascale Pomey, Mike Benigeri, Anne-Marie Cloutier, Marc Dorais, Josiane Courteau, Mireille Courteau, Stéphanie Plante, Pierre Cambon, Annie Giguère, Isabelle Leroux, Danielle St-Laurent, Denis Roy, Jaime Borja, André Néron, Geneviève Landry, Jean-François Ethier, Roxanne Dault, Marc-Antoine Côté-Marcil, Pier Tremblay, Sonia Quirion

**Affiliations:** 1 McGill Observatory on Health and Social Services Reforms, Montreal, Canada; 2 Department of Epidemiology, Biostatistics, and Occupational Health, McGill University, Montreal, Canada; 3 Centre for Research on Planning and Development (CRAD), Laval University, Quebec, G1V 0A6 Canada; 4 Evaluation Platform on Obesity Prevention, Quebec Heart and Lung Institute Research Center, Quebec, G1V 4G5 Canada; 5 Department of Sociology, McGill University, Montreal, Canada

## Abstract

**Background:**

Social inequalities in complications associated with diabetes mellitus persist. As a primary care sensitive condition (PCSC), this association could be related to differential access to primary care. Our objectives are to establish a typology of care trajectories following a new diagnosis, and to explore social determinants of trajectories.

**Methods:**

We used the TorSaDe (The Care Trajectories-Enriched Data) cohort, which links Canadian Community Health Survey respondents to health administrative data. Care trajectories were mapped over a two-year period following a new diagnosis and analysed using state sequence and clustering methods. Associations between individual and geographic characteristics with trajectory types were assessed with multinomial logistic regression.

**Results:**

Three trajectories were identified: Regular Family Physician (FP) Predominant, Specialist Physician Predominant, and Few Services. With Regular FP as the reference, males had higher odds of experiencing the Few Services trajectory, higher education was associated with higher odds of both the Few Services and the Specialist trajectories, and immigrants had higher odds of the Specialist trajectory. Diagnoses in a physician’s office, as opposed to in hospital, were associated with higher odds of the Regular FP trajectory.

**Conclusions:**

The Regular FP trajectory most closely aligns with the management principles of the PCSC approach. We did not find strong evidence of social status privileging access to this trajectory. However, the association with location of diagnosis suggests that efforts to ensure patients diagnosed in hospital are well linked to a regular family physician for follow up may help to reduce unnecessary specialist use and meet PCSC goals.

**Supplementary Information:**

The online version contains supplementary material available at 10.1186/s12913-021-07450-9.

## Introduction

The global prevalence of diabetes has been on the rise for the past several decades [1]. The potential over time for damage to the heart, blood vessels, eyes, kidneys, and nerves necessitates long-term clinical follow up and care [1]. The *care trajectory* concept describes the sequence of healthcare use over time [2]. Understanding the pattern of care set in motion by the diagnosis of diabetes will support the identification of care trajectories that minimize unnecessary or inappropriate services while maximizing health outcomes, and as such has important implications for health system design and for improving patient experiences.

### Primary care for quality diabetes care

Good control of diabetes (maintaining an average HbA1c below 6.5%, 48 mmol/mol) in the first year following a diabetes diagnosis has been associated with reduced risk of complications and death 10 years later, even after adjusting for glycemic control after the first year [3]. This “legacy effect” of early control highlights the importance of ensuring patients are appropriately connected to support and service resources following diagnosis. Considered a primary care sensitive condition (PCSC) [4], effective management is highly contingent on timely access to quality primary care, and notably continuity of care (CoC), which is characterised by an ongoing, cooperative relationship between a patient and their physician-led care team [5]. Higher CoC has been associated with lower risk of preventable hospitalization following a new diagnosis of diabetes [6].

### Socioeconomic disparities in care

Lower socioeconomic status, as well as sociodemographics including education level, sex, and immigrant-status, have been linked to poorer diabetes outcomes – notably through health behaviours, but also poorer access to health care (even in settings with universal insurance) and quality of care [7–10]. These studies often use summary indicators of healthcare access, which can limit information about the order and timing of care in the trajectory. An understanding of these elements is relevant to improving interventions in diabetes management, considering the “legacy effect” of early control of diabetes. Longitudinal studies of the patterns of healthcare use could play a critical role in our understanding of how inequalities in diabetes care arise – and hence, where interventions could limit their progression into inequalities in avoidable morbidity.

With this in mind, we aimed to explore care trajectories defined by states of care as ascribed to interactions across the health care system, in order to better represent the order, timing, and continuity of care. Our main objectives were to assess patterns of care trajectories in the 2 years following a diabetes diagnosis, and to explore social determinants of these patterns.

## Methods

### Study design

This was a retrospective cohort study in the province of Quebec, Canada. The study was approved by the *Commission d’accès à l’information, l’Institut de statistique du Québec*, and McGill’s Research Ethics Board. All methods were performed in accordance with the relevant guidelines and regulations. The need for informed consent was waived by McGill’s Research Ethics Board as this was a retrospective analysis of de-identified administrative data.

### Data sources

We used the Care Trajectories – Enriched Data (TorSaDE) cohort for this study, which is a linkage of Canadian Community Health Survey (CCHS) and health administrative data. Phase 1 of the cohort consists of a sample of 61,083 consenting Quebec CCHS respondents (from survey years 2007 to 2012) and 19 years of health administrative data [11].

Briefly, the CCHS is a comprehensive population-based cross-sectional health survey with self-reported information on health status and health determinants. As our goal was to make inference about the TorSaDE cohort, and not the Canadian population, we opted not to conduct weighted analyses. This decision was in alignment with other studies using linked survey and administrative data in similar ways [12, 13].

As elsewhere in Canada, Quebec has a universal health insurance program, covering residents of the province for all medically necessary physician and hospital services. Health administrative data correspond to all physician visits reimbursed on a fee-for-service basis, and all hospital visits (1996 to 2016). Data include the date, diagnosis code, patient and physician identifiers, and service descriptors.

To identify a subcohort of individuals with incident diabetes, we used a validated algorithm of two physician visits or one hospitalization with a diabetes code in a 2 year period [14]. To ensure our subcohort represented people with incident cases, we restricted the subsample to cases without any previous diabetes code registered in the 5 years preceding the incident date, and excluded females with pregnancy-related events within 5 months of the diagnosis [15]. Our subcohort included adults aged 20 years and older, to eliminate the period of transition from pediatric to adult programs that occurs at age 18 [16].

### Care trajectories

The index date was the day of diabetes diagnosis, as identified by the algorithm [14]. We followed the care trajectory for 2 years, beginning on the day after diagnosis.

To create the care trajectories, we identified all physician and hospital encounters in the follow-up period for each individual. We included any visit diagnosis code, not solely diabetes-related visits, as we were interested in total healthcare use. Our care trajectories were organized by five states which were defined based on our review of the data and interpretation of important distinctions in care: regular family physician care, new family physician care, specialist care, emergency department or hospital care, and no health service. We defined a regular family physician (FP) as one with whom ≥50% of the individual’s FP visits in the preceding 365 days had occurred. A new FP was defined as any with whom < 50% of these visits had occurred. An individual who had seen a physician on an outpatient basis was considered to be under the care of that physician for 90 days or until the next health care encounter to align with recommendations for follow up every 3 months for less well-controlled diabetes [17]. If no further health care encounter occurred before the expiration of the 90-day period, the individual was considered to not be under active care of a provider (“no health service”). In the present analysis, emergency department visits were grouped with hospitalizations due to relatively small numbers in these categories and the conceptual similarity in institution-based care states.

The trajectories initially lasted 730 days, with one state coded for each day based on the conditions described above. However, given the precondition that a diagnosis occurs in office or hospital, i.e., in an active state of care, the state of no health service only began to appear in the trajectories at day 90, causing a clear shift in the distribution of care states. We thus opted to begin our analysis of trajectories from day 91 to eliminate this issue. Sensitivity analyses showed the substantive results were unchanged. As such, the length of trajectories for this analysis is 641 days.

### Variables

Age was recorded in the CCHS and recalculated at the start of the trajectory period. For social demographic measures, we used education level as a proxy for socioeconomic status, because this was a time-invariant measure. Education was categorized as ‘No high school diploma, ‘High school diploma’, ‘Post-secondary diploma’, ‘University degree’. We also explored immigration status, recalculating the time since immigration to the start of the trajectory.

A Combined Charlson and Elixhauser Comorbidity index [18] using Schneeweiss weights [19] was calculated for the year prior to the start of the care trajectory, to adjust for individual-level baseline comorbidity differences. The index is the result of the sum of weights derived from 30-day mortality predictions for each identified condition [18], with a higher index indicating a higher morbidity. Place of diagnosis was based on whether the incidence algorithm flagged a case through physician visit (considered to be in office) or hospitalization (considered to be in hospital).

Geography was explored with a four-category zone variable (census metropolitan area (CMA) of Montreal; other CMA in the province; census agglomeration (CA); rural areas) that distinguished the largest urban centre of Montreal from other urban centres, smaller towns, and rural areas.

### Analysis

The analysis was conducted in three steps. First, state sequence analysis was used to analyze the trajectories [20]. This approach takes every pair of trajectory sequences and applies a sequence alignment algorithm to derive a matrix of dissimilarity. We used the dynamic hamming distance (DHD) alignment algorithm (which does not use an indel cost) [21], with the substitution matrix based on time-varying state transitions. We opted to use the DHD algorithm as optimal matching was too computationally demanding; additionally, this approach was theoretically sound as DHD privileges the order and timing of events, in contrast to optimal matching which prioritizes the occurrence of states [22]. In our application, we assumed that differences in order and timing of the states would be meaningful.

In the second step, cluster analysis was performed on the dissimilarity matrix to group similar trajectories using an agglomerative hierarchical clustering procedure and Ward linkage method. The number of clusters was selected by visually inspecting the inertia curve and the clustering dendrogram and then assessing the meaningfulness of the resulting groups (see supplementary Figs. 1 and 2). After determining the number of clusters that best represented our data, we described the distribution of individuals assigned to each cluster.

As our final step, we assessed the relevance of covariates in predicting membership in each trajectory group using multivariable multinomial logistic regression models, given the polytomous outcome.

## Results

### Descriptive

Our subcohort consisted of 4308 individuals with an incident diagnosis of diabetes, 49% of which (*n* = 2112) were female (Table 1). The mean age was 62 years, 38% did not have a high school diploma, and 8% were immigrants. Three-quarters were diagnosed in a physician office setting, and 29% lived in Montreal, while one-third (33%) lived in a rural location (Table 1). There was very little attrition during the study period, but the records of 130 individuals were removed due to death during follow-up. These deaths predictably occurred among older people (mean age 74 years, standard deviation 10.2), with higher pre-trajectory comorbidities (mean Combined Comorbidity Index score of 6.7, standard deviation 5.2, compared to a mean score of 0.9, standard deviation 2.2, overall for the remaining cohort).

**Table 1:** Distribution of patient characteristics by trajectory group

Characteristic		Total sample (***N*** = 4308)	Regular GP (***n*** = 1854; 43%)	Specialist predominant (***n*** = 1746; 41%)	Few services (***n*** = 708; 16%)
Age	Mean (SD)	61.6 (12.5)	62.1 (11.9)	62.5 (12.7)	58.1 (12.9)
Age groups	20–40	240 (6%)	75 (4%)	105 (6%)	60 (8%)
	41–60	16,778 (39%)	743 (40%)	591 (34%)	344 (49%)
	61–70	1325 (31%)	558 (30%)	574 (33%)	193 (27%)
	71–80	811 (19%)	372 (20%)	365 (21%)	74 (10%)
	80+	254 (6%)	106 (6%)	111 (6%)	37 (5%)
Combined Comorbidity Index in the year before diagnosis	Mean (SD)	0.87 (2.2)	0.6 (1.9)	1.23 (2.5)	0.60 (2.1)
Education	Some high school education	1627 (38%)	779 (42%)	625 (36%)	223 (31%)
	High school diploma	588 (14%)	249 (13%)	244 (14%)	95 (13%)
	College/CEGEP	1572 (36%)	644 (35%)	636 (36%)	292 (41%)
	University degree	481 (11%)	163 (9%)	227 (13%)	91 (13%)
	Missing	40 (1%)	19 (1%)	14 (1%)	7 (1%)
Sex	Female	2112 (49%)	921 (50%)	903 (52%)	288 (41%)
	Male	2186 (51%)	923 (50%)	843 (48%)	420 (59%)
Immigrant	yes	328 (8%)	104 (6%)	174 (10%)	50 (7%)
	no	3977 (92%)	1748 (94%)	1571 (90%)	658 (93%)
	missing		2 (0%)	1 (0%)	0 (0%)
Years since immigration	Mean (SD)	34.1 (17.7)	35.6 (16.6)	34.0 (18.3)	30.2 (18.7)
Service use during trajectory Mean (SD)	Regular GP	8.9 (7.5)	11.9 (8.8)	7.7 (5.6)	3.7 (2.8)
	Specialist visits	11.5 (14.9)	7.0 (6.7)	19.1 (19.8)	4.4 (4.3)
	Hospital days	20.6 (34.8)	18.9 (34.0)	23.5 (38.8)	17.8 (24.0)
	Emergency visits	4.2 (5.2)	3.6 (3.7)	5.2 (6.8)	3.4 (3.4)
	New GP visits	4.2(4.7)	3.5 (3.5)	5.2 (5.9)	3.4 (3.3)
Place of diagnosis	office	3259 (76%)	1528 (82%)	1185 (68%)	546 (77%)
	hospital	1049 (24%)	326 (18%)	561 (32%)	162 (23%)
Residential Area (at start of trajectory)	CMA of Montreal^a^	1269 (29%)	460 (25%)	614 (35%)	195 (28%)
	Non-Montreal CMA	868 (20%)	404 (22%)	339 (19%)	125 (18%)
	Census agglomeration (CA)	731 (17%)	335 (18%)	265 (15%)	131 (19%)
	Rural area	1420 (33%)	647 (35%)	526 (30%)	247 (35%)
	missing	20 (0%)	8 (0%)	2 (0%)	10 (1%)

^a^*CMA* Census Metropolitan Area

**Fig. 1:**
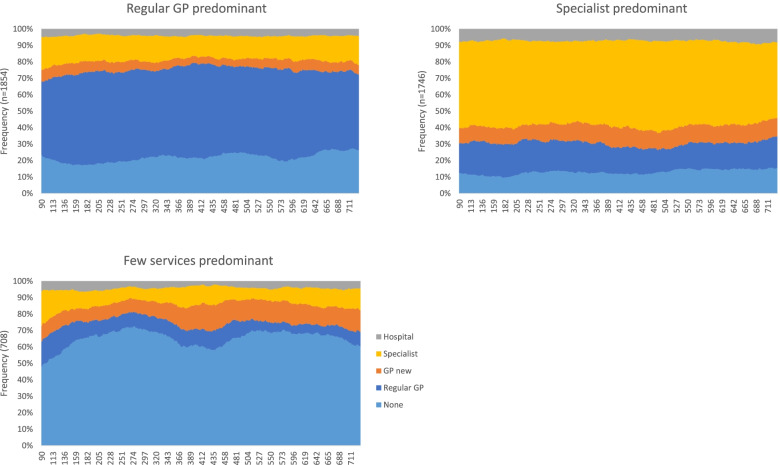
Sequence analysis trajectory groups showing the daily distribution of respondents in each care state over time (the x-axis represents days)

The sequence analysis and clustering procedure resulted in three distinct groups (see supplementary Figs. 1 and 2 for the dendrogram and inertia curves, respectively, which we named based on the predominant care states (Fig. 1).

**Fig. 2:**
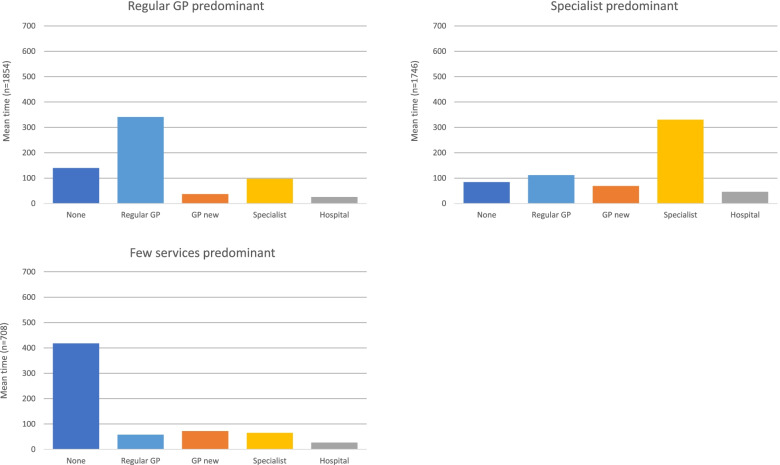
Average number of days spent in each care state by trajectory type

The largest cluster (43% of the sample) formed the pattern we call Regular FP Trajectory. Individuals following this trajectory type spent an average of 341 days (out of the 641, i.e., 53% of the trajectory days) in the regular family physician care state, and a small proportion of time in the hospital and emergency care state (mean = 26 days, i.e., 4% of trajectory days) (Fig. 2).

The next largest group (41%) followed a pattern we termed Specialist Trajectory. Individuals in this group spent an average of 330 days (51%) in the specialist care state. They spent the most time, on average, of the three groups in the hospital and emergency care state (mean = 46 days; 7%).

Finally, 16% of the sample followed a pattern we call Few Services Trajectory, spending an average of 418 days (65% of trajectory days) in a state with no services. This group had the lowest specialist and regular family physician involvement.

Respondents following the Specialist Trajectory spent a similar amount of time as the Few Services Trajectory members in the new family physician care state (mean = 69 and 72 days, respectively), but nearly twice as much time as those members in the regular family physician care state (112 days vs. 58 days). People in the Regular FP Trajectory spent about the same amount of time in the hospital and emergency care state as the Few Services Trajectory members (mean = 26 days and 27 days respectively) (Fig. 2).

While the distribution of men and women following the Regular FP Trajectory was evenly split, there was a higher proportion of men in the Few Services Trajectory, and a higher proportion of women in the Specialist Trajectory (Table 1). The Few Services and Specialist Trajectories had a higher proportion of people with a university degree. There was a higher proportion of immigrants in the Specialist than in the other trajectory groups. There were more younger people and fewer older people in the Few Services Trajectory. People living in the urban centre of Montreal at diagnosis had higher representation in the Specialist Trajectory.

## Model results

### Specialist vs. family physician

**Fig. 3:**
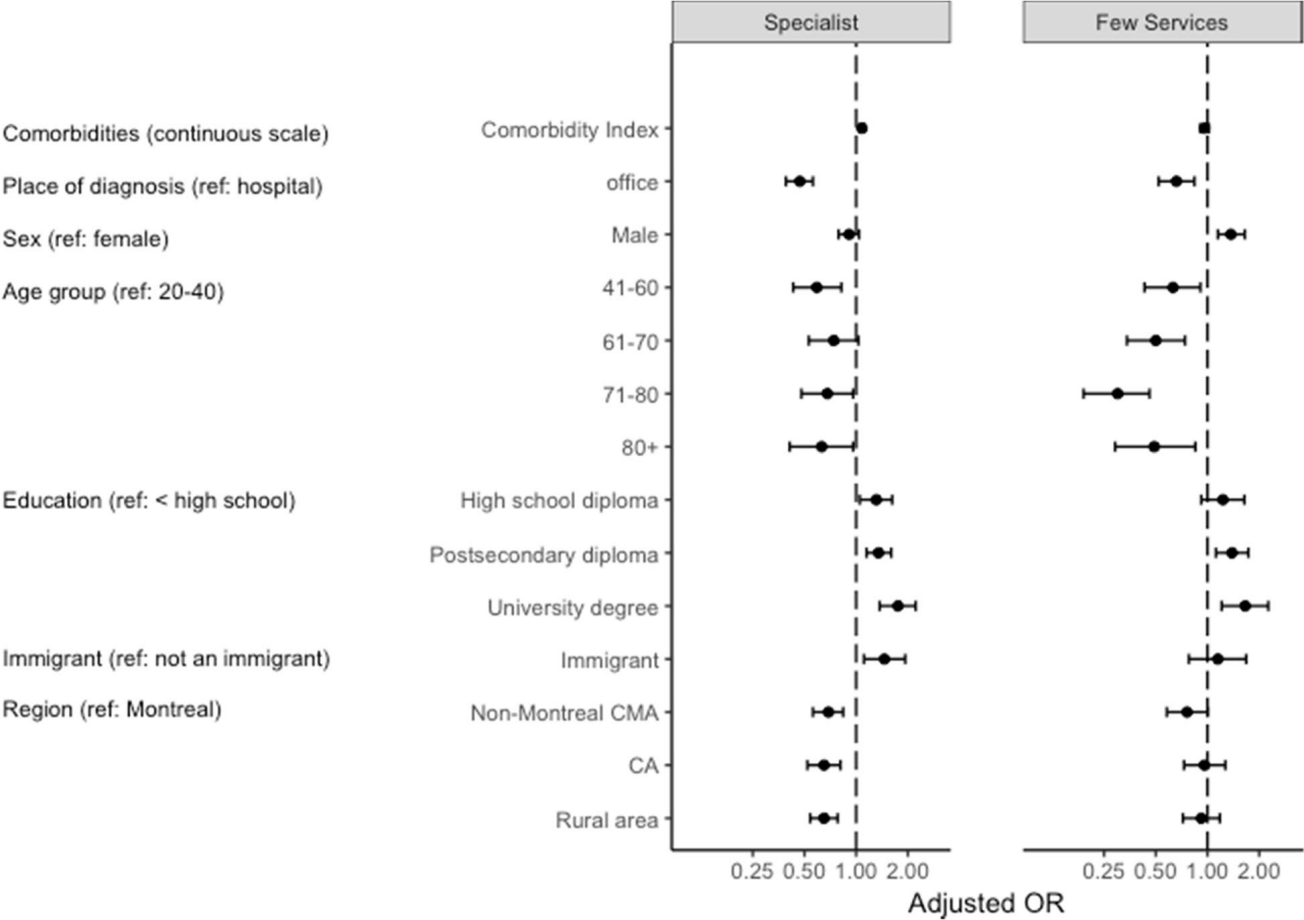
Multinomial logistic regression model of factors associated with trajectory group membership, in reference to the Regular Family Physician Trajectory. CMA = Census Metropolitan Area; CA = Census Agglomeration

All of the model results presented are adjusted for all other covariates. Higher comorbidity was associated with higher odds of membership in the Specialist as compared to the Regular FP Trajectory (adjusted odds ratio, aOR 1.08, 95% CI 1.05–1.12) (Fig. 3; see also supplementary Table 1). Diabetes diagnosis in a physician’s office, as compared to in hospital, was associated with lower odds of membership in the Specialist Trajectory (aOR 0.47, 95% CI 0.39–0.56). Higher levels of education were associated with higher odds of membership in this trajectory, as compared to no high school diploma (aOR 1.31, 95% 1.05–1.62; OR 1.35, 95% CI 1.15–1.60; OR 1.75, 95% CI 1.37–2.22 for high school diploma, college/CEGEP, and university degree, respectively). Being an immigrant was associated with higher odds of membership in this trajectory (aOR 1.46, 95% CI 1.11–1.93). Living outside of the Montreal urban area was associated with lower odds of following this Specialist Trajectory (aOR 0.69, 95% CI 0.56–0.84; OR 0.65, 95% CI 0.52–0.81; OR 0.65, 95% CI 0.54–0.78 for non-Montreal CMA, CA, and rural area, respectively).

### Few services vs. family physician

Being diagnosed in an office as compared to in hospital was associated with lower odds of following the Few Services Trajectory, compared to the Regular FP Trajectory (aOR 0.66, 95% CI 0.52–0.84). Males had higher odds of membership in the Few Services Trajectory than females (aOR 1.37, 95% CI 1.15–1.65). Increasing age groups had lower odds of membership in this trajectory (compared to ages 20–40: aOR 0.63, 95% CI 0.43–0.91 for ages 41–60; aOR 0.50, 95% CI 0.34–0.74 for ages 61–70; aOR 0.30, 95% CI 0.19–0.46 for ages 71–80; and aOR 0.49, 95% CI 0.29–0.85 for ages 80+). Having a college/CEGEP or university degree, compared to no high school diploma, was positively associated with membership in the Few Services Trajectory (aOR 1.39, 95% CI 1.12–1.73; OR 1.66, 95% CI 1.21–2.26 respectively). While living in an urban area other than Montreal was associated with lower odds of membership in the Few Services Trajectory, this was borderline non-significant (aOR 0.76, 95% CI 0.58–1.00). Other areas of residence showed no differences in the few services membership pattern compared to residence in Montreal.

## Discussion

Our study aimed to identify a typology of care trajectories following diabetes diagnosis and to explore sociodemographic variables associated with trajectory-type membership. From this analysis, we describe three distinct patterns of all-cause health care utilization in the first 2 years following a diagnosis of diabetes in a cohort of people in Quebec. Forty-three percent of our sample followed a trajectory defined predominantly by regular FP care. Another 41% followed a trajectory comprising mostly specialist care with some involvement of regular FPs as well as “new” (non-regular) FPs. The rest (16%) followed a pattern characterized by few services overall. Those with few services spent an approximately equivalent amount of time in the hospital and emergency care state as those individuals following the Regular FP Trajectory.

Various methods have previously been used to explore and describe different patterns in healthcare utilization. One recent study measured care seeking as irregular provider contact, regular specialized care, and regular generalized care for diabetes based on yearly care patterns [23]. Using group-based trajectory modeling over an 11-year period, they found seven trajectories (persistent irregular use, generalized to irregular, irregular to generalized, persistent generalized, generalized to specialized, specialized to generalized, and persistent specialized); membership to which differed by age, SES, and residential location [23]. Another approach applied a tailored state sequence analysis to study where a service was undertaken, by which specialist, and for which diagnosis among a cohort of patients with chronic obstructive pulmonary disease followed for 1 year [24]. Five trajectory types, corresponding to low healthcare utilization, moderate healthcare utilization, and three high utilization groups with predominantly respiratory diagnoses, cardiovascular diagnoses, and other diagnoses, respectively [24]. Our analysis took a slightly different approach in assigning care states a lasting duration. Nevertheless, our results indicate a low utilization group, a moderate utilization group dominated by regular family physician care, and a higher utilization group dominated by specialist care.

Our results suggest a path dependence in access to care. Individuals in the Specialist Trajectory spent nearly twice as long overall in the hospital and emergency care state. It is conceivable that patients connect with specialists during a hospital encounter, and then potentially bypass the family physician or otherwise have direct follow ups with specialists [25]. Indeed, diagnosis in a physician’s office (i.e., outside of hospital) was associated with lower odds of following the Specialist Trajectory than diagnosis in hospital, as compared to the Regular FP Trajectory. This may reflect differences in the pre-diagnosis trajectory, suggested also by the fact that comorbidities were statistically significantly higher in the Specialist Trajectory than the Regular FP Trajectory. People with the Few Services Trajectory showed no significant differences in comorbidities compared to those with the Regular FP Trajectory.

The second part of our objective was to investigate inequalities in care trajectories. We have argued that a trajectory favouring continuity of care with a regular FP is optimal, as it most closely represents the recommended chronic care management model of PCSCs [4]. While we did observe some socially patterned differences in the odds of following different types of trajectories, we did not find strong evidence of social status privileging access to the Regular FP Trajectory. Immigrants and people with higher education had higher odds of following the Specialist Trajectory as compared to the Regular FP Trajectory. People with higher education also had higher odds of following the Few Services trajectory, which may seem a counterintuitive finding. This result could also be reflective of other dynamics including less frequent visits to a FP due to higher participation in diabetes management programs run outside the family physician’s clinic, or a higher perceived sense of control over condition management recommendations. However, a recent Canadian study has found that people with higher education reported greater difficulties in accessing health care, suggesting that there may be further explanations to explore. After accounting for comorbidities, older people had lower odds of following the Specialist Trajectory, as well as lower odds of following the Few Services Trajectory, likely because they already had access to a family physician.

Men were more likely than women to follow the Few Services Trajectory, as compared to the Regular FP Trajectory, a finding that is supported by studies showing that men are less likely to visit an FP compared to women [26].

Previous studies have shown a higher rate of specialist visits with higher income and education levels in Canada, even after adjusting for health care need, which suggests the potential influence of these factors on the likelihood of a referral from primary care [27]. In the present study, we found that residents of areas outside of the large urban centre of Montreal were also less likely to follow the Specialist Trajectory. This could be attributable to geographic disparities in access to a family physician – the proportion of people in Montreal with a regular family physician is lower than elsewhere in the province [28] – or to a supply effect of the higher concentration of specialists in the urban centre of Montreal [29].

In our analysis, immigrants were more likely to follow the Specialist Trajectory than non-immigrants, as compared to the Regular FP Trajectory. Immigrants may be less likely to have access to a regular family physician [30], although other studies have not demonstrated this difference [31]. Of note, we found that immigrants were no more likely to follow the Few Services Trajectory than non-immigrants after adjusting for place of residence. This suggests that the concentration of immigrants in a large urban area (Montreal, QC) was not driving the effect. However, grouping all immigrants into one category undoubtedly glosses over nuances.

### Strengths and limitations

This study uses a novel cohort in Quebec of survey participants linked to administrative data, which allows richer socio-demographic data than is normally available in administrative data only.

This analysis was not designed to test causal differences in following different trajectory types. However, the socio demographic factors we explored here are all relatively time invariant and would be determined prior to the start of the trajectory. We have not yet assessed outcomes associated with trajectory types membership, which limits our ability to draw conclusions about their effectiveness. This will be the basis of our subsequent work in this area.

We used education instead of income as a proxy measure of SES; as the CCHS interview could occur at varying intervals around the start of the trajectory, we did not have an income measure consistent with the start of the trajectory. We thus opted for measures that would be fixed in time. In support of this decision, we note that in a universal care system with a single government payer, income would be less expected to impact access to or utilization of care. Education level, on the other hand, may influence health literacy and patients’ abilities to navigate the health system. Indeed, previous studies have found education to show stronger associations with health service utilization than income [32].

We defined a regular FP as the one providing at least half of an individual’s family medicine visits in the past year. While stricter definitions have been used [33], we still see important differences emerge with our broader approach. Importantly though, we are lacking information on registration with a *groupe de médecin de famille* (family medicine group, GMF), so we cannot rule out that the “non-regular” FP visits are not with another physician in an individual’s GMF. GMFs were designed to improve continuity of care, even when an individual is not able to see their own regular physician.

Finally, we could not distinguish between type 1 and 2 diabetes with our algorithm; however, given our age criteria of individuals 20 years old and above it is reasonable to assume that our cohort is primarily composed of individuals with type 2. Type 1 diabetes accounts for 5–10% of all diabetes diagnoses, and occurs mostly in children [34]. Our approach to the analysis assessed all-cause health care utilization. It will be important in subsequent work to identify the intensity of diabetes-specific utilization within and among trajectory groups, and to distinguish this from other primary diagnoses associated with healthcare encounters.

Our approach to measure healthcare use maintains daily granularity. Alternatives include measuring use in wider time blocks, such as monthly. However, this would necessitate defining a hierarchy to account for the potential of multiple care states during the time period. As we were equally interested in the presence of each state, we wanted to avoid making hierarchical determinations. However, this daily approach may have been too granular to allow for changing longitudinal trends in care states to be detected (such as shifts from specialist to family physician care, or from new to regular family physician care, over time).

The benefit of sequence analysis is the ability to simultaneously consider the evolution of care interactions over time, without focusing explicitly on transitions between states – the trajectories are analysed as a static object, while allowing for dynamic patterns within [35]. This is a significant benefit in chronic condition health care utilization research, where transitions between care states may be of less interest than the overall pattern of care.

### Potential policy implications

The medical home model of health care prioritizes strong connections to a primary care provider or team, with support of specialist consultants as necessary. The Regular FP care trajectory that we used as reference fits well within this model and with that of PCSCs, which stipulates that most treatment, monitoring, and support for diabetes can and should occur in the primary care setting [17, 36]. To note, this care trajectory is not only deemed optimal for the patient, but also for the system, as it prevents unnecessary, more costly, and potentially invasive care [4].

Our analysis identified a pattern of specialist-predominant services that would generally not conform to the PCSC model. In fact, specialist care is not uniformly associated with better outcomes following a diabetes diagnosis, despite being associated with the use of appropriate diabetes-specific treatments [37]. Efforts could be encouraged to link patients diagnosed in hospital with suitable family physicians willing to take charge of the chronic issue management, if a family physician is not already assigned. In Quebec, this could be implemented through the *guichet d’accès à un médecin de famille* (GAMF), which is a centralized intake and waitlist system for individuals seeking a family physician [38].

## Conclusion

This analysis of a survey-based cohort linking to health administrative records found three relatively stable trajectory types to be prevalent among adults with a new diagnosis of diabetes. Further understanding how sociodemographic and other factors relate to a patient’s experience with following typical trajectories of care will serve to identify ways to enhance the patient’s experience, the quality and appropriateness of care, and ultimately reinforce the ability to steer patients towards care trajectories best aligned with the PCSC care approach.

## Supplementary Information

**Additional file 1.**

## Data Availability

The data that support the findings of this study are available from the *Institut de la statistique du Québec* but restrictions apply to the availability of these data, which were used under license for the current study, and so are not publicly available. Data are however available from the authors upon reasonable request and with permission of ISQ, authorization from the CAI, and other appropriate approvals from relevant data stewards.
