# Supplementary material for: Exploring social inequalities in healthcare trajectories following diagnosis of diabetes: a state sequence analysis of linked survey and administrative data
Source: BMC Health Serv Res. 2022 Jan 31;22:131. doi: 10.1186/s12913-021-07450-9 (PMC8805244; doi:10.1186/s12913-021-07450-9)
Supplement: Supplementary file 1 — Additional file 1. [file 12913_2021_7450_MOESM1_ESM.docx]

# Supplementary material


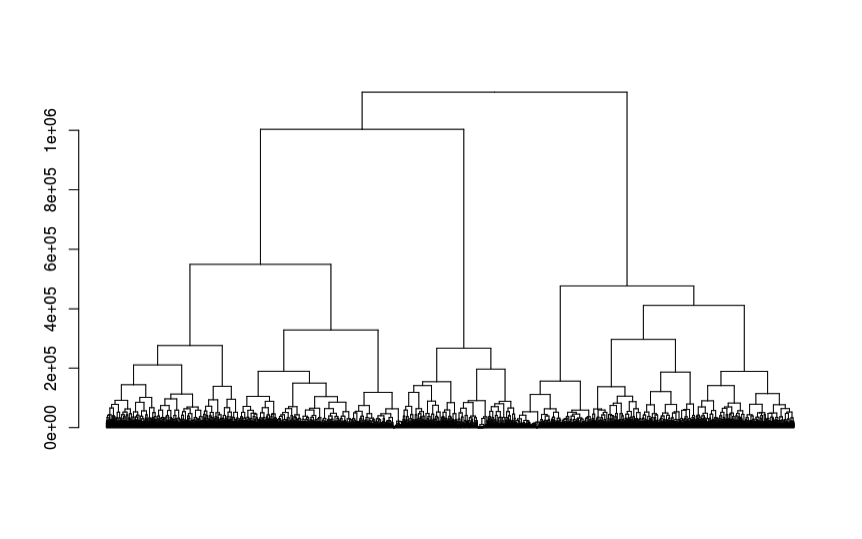


Figure S 1: Dendrogram of sequence analysis results


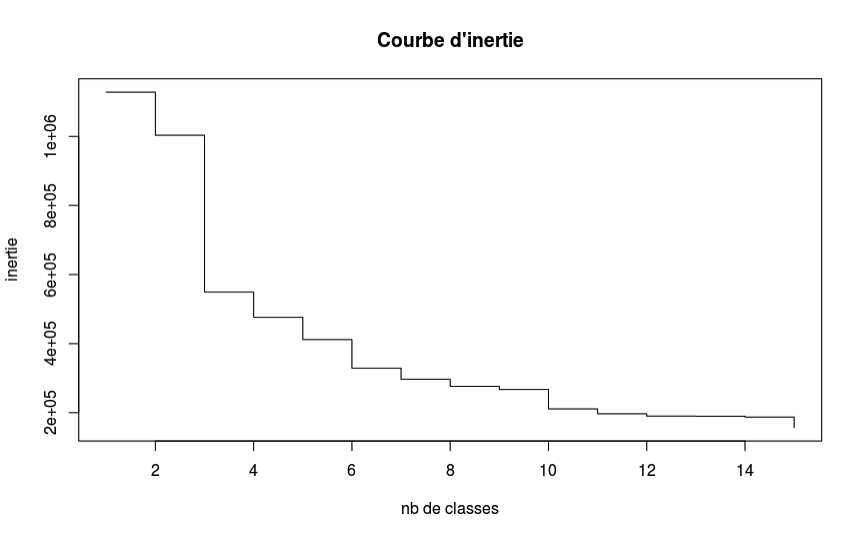


Inertia

Number of clusters

Figure S 2: Inertia curve of sequence analysis results.

Table S 1: Multinomial logistic regression model results

|  |  | **OR (95% CI)** | |
| --- | --- | --- | --- |
|  |  | Specialist | Few Services |
| (Intercept) |  | 2.57 (1.75 - 3.79) | 0.75 (0.46 - 1.20) |
| Age group  (ref: 20-40) | 41-60 | 0.59 (0.43 - 0.82) | 0.63 (0.43 - 0.91) |
|  | 61-70 | 0.74 (0.53 - 1.03) | 0.50 (0.34 - 0.74) |
|  | 71-80 | 0.68 (0.48 - 0.96) | 0.30 (0.19 - 0.46) |
|  | 80+ | 0.63 (0.41 - 0.96) | 0.49 (0.29 - 0.85) |
| Sex (ref: female) | Male | 0.91 (0.79 - 1.04) | 1.37 (1.15 - 1.65) |
| Education  (ref: some high school education) | High school diploma | 1.31 (1.05 - 1.62) | 1.23 (0.92 - 1.64) |
|  | College/CEGEP | 1.35 (1.15 - 1.60) | 1.39 (1.12 - 1.73) |
|  | University degree | 1.75 (1.37 - 2.22) | 1.66 (1.21 - 2.26) |
| Immigrant status  (ref: not an immigrant) | Immigrant | 1.46 (1.11 - 1.93) | 1.15 (0.78 - 1.68) |
| Comorbidity index |  | 1.08 (1.05 - 1.12) | 0.96 (0.91 - 1.02) |
| Location of diagnosis  (ref: hospital) | Physician's office | 0.47 (0.39 - 0.56) | 0.66 (0.52 - 0.84) |
| Region  (ref: Montreal) | Other urban | 0.69 (0.56 - 0.84) | 0.76 (0.58 - 1.00) |
|  | 10,000 - 100,000 habitants | 0.65 (0.52 - 0.81) | 0.96 (0.73 - 1.27) |
|  | small village/rural | 0.65 (0.54 - 0.78) | 0.92 (0.72 - 1.18) |
